# High Level of Viral Suppression and Low Switch Rate to Second-Line Antiretroviral Therapy among HIV-Infected Adult Patients Followed over Five Years: Retrospective Analysis of the DART Trial

**DOI:** 10.1371/journal.pone.0090772

**Published:** 2014-03-13

**Authors:** Cissy Kityo, Diana M. Gibb, Charles F. Gilks, Ruth L. Goodall, Ivan Mambule, Pontiano Kaleebu, Deenan Pillay, Ronnie Kasirye, Peter Mugyenyi, A. Sarah Walker, David T. Dunn, on behalf of the DART Trial Team

**Affiliations:** 1 Joint Clinical Research Centre, Kampala, Uganda; 2 MRC Clinical Trials Unit at UCL, London, United Kingdom; 3 School of Population Health, University of Queensland, Australia; 4 Infectious Diseases Institute, Mulago, Uganda; 5 MRC/UVRI Uganda Research Unit on AIDS, Entebbe, Uganda; 6 University College London, London, United Kingdom; Massachusetts General Hospital, United States of America

## Abstract

In contrast to resource-rich countries, most HIV-infected patients in resource-limited countries receive treatment without virological monitoring. There are few long-term data, in this setting, on rates of viral suppression or switch to second-line antiretroviral therapy. The DART trial compared clinically driven monitoring (CDM) versus routine laboratory (CD4/haematology/biochemistry) and clinical monitoring (LCM) in HIV-infected adults initiating therapy. There was no virological monitoring in either study group during follow-up, but viral load was measured in Ugandan participants at trial closure. Two thousand three hundred and seventeen (2317) participants from this country initiated antiretroviral therapy with zidovudine/lamivudine plus tenofovir (n = 1717), abacavir (n = 300), or nevirapine (n = 300). Of 1896 (81.8%) participants who were alive and in follow-up at trial closure (median 5.1 years after therapy initiation), 1507 (79.5%) were on first-line and 389 (20.5%) on second-line antiretroviral therapy. The overall switch rate after the first year was 5.6 per 100 person-years; the rate was substantially higher in participants with low baseline CD4 counts (<50 cells/mm^3^). Among 1207 (80.1%) first-line participants with viral load measured, HIV RNA was <400 copies/ml in 963 (79.8%), 400–999 copies/ml in 37 (3.1%), 1,000–9,999 copies/ml in 110 (9.1%), and ≥10,000 copies/ml in 97 (8.0%). The proportion with HIV RNA <400 copies/ml was slightly lower (difference 7.1%, 95% CI 2.5 to 11.5%) in CDM (76.3%) than in LCM (83.4%). Among 252 (64.8%) second-line participants with viral load measured (median 2.3 years after switch), HIV RNA was <400 copies/ml in 226 (89.7%), with no difference between monitoring strategies. Low switch rates and high, sustained levels of viral suppression are achievable without viral load or CD4 count monitoring in the context of high-quality clinical care.

**Trial Registration:**

ISRCTN13968779

## Introduction

Most resource-limited countries have adopted a public health approach to anti-retroviral therapy (ART) for the treatment of HIV infection in which the public sector provides a single first-line regimen, with alternative substitute drugs as required, and a standard second-line therapy for those who fail first-line [1], [2]. The limited availability of laboratory tests requires the flexible use of routine viral load or CD4 count monitoring to detect treatment failure according to local circumstances [3]. In contrast, the care of HIV-infected patients in resource-rich countries is highly individualised, including the regular measurement of viral load to check that current ART is successfully inhibiting viral replication.

When viral load is not routinely monitored some patients may experience periods of prolonged undetected viraemia, which has several potential negative consequences. First, long delays in switching therapy may place the patient at increased risk of opportunistic infections although regular CD4 monitoring should mitigate against this [4]. Second, evidence has emerged that viraemia per se may have adverse chronic effects, possibly via elevated immune activation [5], [6]. Third, extensive drug resistance may develop, thereby compromising the virological efficacy of second-line ART if there is cross-resistance between drugs used in first-line and second-line regimens. This also carries a public health threat in that the transmission of resistant viruses could increase and thus eventually limit the effectiveness of first-line ART [7]. Fourth, CD4 count is generally weakly predictive of virological failure [8], although the association is stronger among patients with clinical symptoms [9]. Finally, it has been suggested that patients' knowledge of their viral load values might help improve adherence to therapy, although randomised evidence is lacking [10]. Based on these considerations, several experts have questioned whether it is ethical to administer ART without viral load monitoring [4], [11]–[14].

However, these concerns clearly need to be balanced against the critical point that in any financially-constrained healthcare system facing static or diminishing funds for HIV/AIDS programmes, resources directed towards laboratory testing mean that fewer patients in need of treatment are able to receive it [2], [15], [16]. Further, routine viral load monitoring results in higher switch rates to more costly second-line ART [17], [18]. Finally, viral load testing is technically complex, making its application in resource-limited settings challenging [19]. Some programmes have found that erroneous results were frequently reported to clinicians, potentially leading to unnecessary ART regimen change or enhanced adherence counselling, and undetected virological failure [19].

The debate on viral load monitoring in resource-limited settings has been conducted with remarkably few relevant data to inform it. Here we report cross-sectional viral load results after five years on ART among Ugandan patients in the DART trial, where clinical management (in particular, switch from first-line to second-line ART) was based on clinical symptoms with or without access to CD4 counts in the absence of real-time viral load monitoring [20].

## Methods

### Study Overview

DART (Development of Antiretroviral Therapy in Africa) was an open randomised trial in ART-naive, symptomatic HIV-infected adults with a CD4 count ≤200 cells/mm^3^, enrolled from three clinical centres in Uganda and one in Zimbabwe between January 2003 and October 2004 [20]. Participants were randomised to clinically-driven monitoring (CDM) or routine laboratory (CD4 cells counts, haematology, and biochemistry tests) plus clinical monitoring (LCM), and followed under these strategies until the end of 2008. DART included two sub-studies of second-line therapy that are pertinent to the current analysis, both of which opened for recruitment in July 2007 and whose populations partly overlapped: OHFS (Optimal HAART Feasibility Study) [21] and SARA (Second-line Antiretroviral Therapy in Africa) [22].

### Viral load measurements

Although there was no real-time viral load monitoring in DART, Ugandan participants were eligible for a viral load test under the national programme shortly after trial closure. However, participants who were enrolled in either OHFS or SARA were ineligible since viral load was measured (retrospectively) at specific time points as part of the protocol of these sub-studies. To increase the number of available measurements and to decrease potential bias, we included viral load results from these two sub-studies that coincided with the testing done within the national programme (January 2009 to April 2009). All viral load assays were done in two centres: Joint Clinical Research Centre, Kampala (Roche Taqman 1.0, lower limit of detection [LLD] = 40 copies/ml) and the Infectious Diseases Institute, Mulago (Roche Amplicor 1.5, LLD = 400 copies/ml). Viral suppression was defined as HIV RNA<400 copies/ml (i.e. the higher of the two LLDs).

### Antiretroviral regimens

First-line ART regimens comprised co-formulated zidovudine (ZDV)-lamivudine (3TC) plus either tenofovir (TDF), abacavir (ABC), or nevirapine (NVP) [20], [23]. Following World Health Organization (WHO) guidelines, the protocol discouraged switching in the first year of therapy. Thereafter, the decision to switch to second-line ART was based on clinical criteria (new/recurrent WHO stage 4 event, WHO stage 3 events at the discretion of the treating physician) in both groups, along with confirmed CD4 count <100 cells/mm^3^ in the LCM group only [20].

All second-line ART regimens included the boosted protease inhibitor (PI), lopinavir/ritonavir (LPV/r). The nucleoside/nucleotide reverse transcriptase inhibitors (NRTIs) and/or non-nucleoside reverse transcriptase inhibitors (NNRTI) prescribed in combination with LPV/r were allocated according to the OFHS randomisation schedule if the individual participated in this sub-study, or otherwise at the discretion of the patient's clinician [21]. In the SARA sub-study, participants who had been on second-line ART for 24 weeks were randomised between continuing their boosted PI-containing regimen or reducing to maintenance boosted PI monotherapy [22].

### Statistical methods

The probability of switching to second-line ART by trial closure was examined by multivariate logistic regression analysis, including a priori selected baseline predictors (i.e. monitoring strategy, age, sex, baseline CD4 count, first-line ART regimen). A simplified version of this model, combining the two triple NRTI regimens (ZDV/3TC/TDF, ZDV/3TC/ABC) and excluding non-significant covariates (P>0.05), was used to estimate absolute probabilities (by converting from odds) of switching for combinations of variables. Logistic regression analysis was used to examine if the absence of a viral load measurement was related to any of the baseline variables or last available CD4 count. Logistic regression analysis, applied separately to participants on first-line and second-line ART, was similarly used to examine predictors of viral suppression at trial closure, adjusting for duration of the specific line of ART. Estimates of the overall prevalence of viral suppression (i.e. combining first-line and second-line regimens) were derived using weighted averages to account for the relative under-representation of participants on second-line ART. All analyses were performed using STATA (version 12.0).

### Ethics statement

The study was approved by the Uganda Virus Research Institute, Entebbe.

## Results

**Figure 1:**
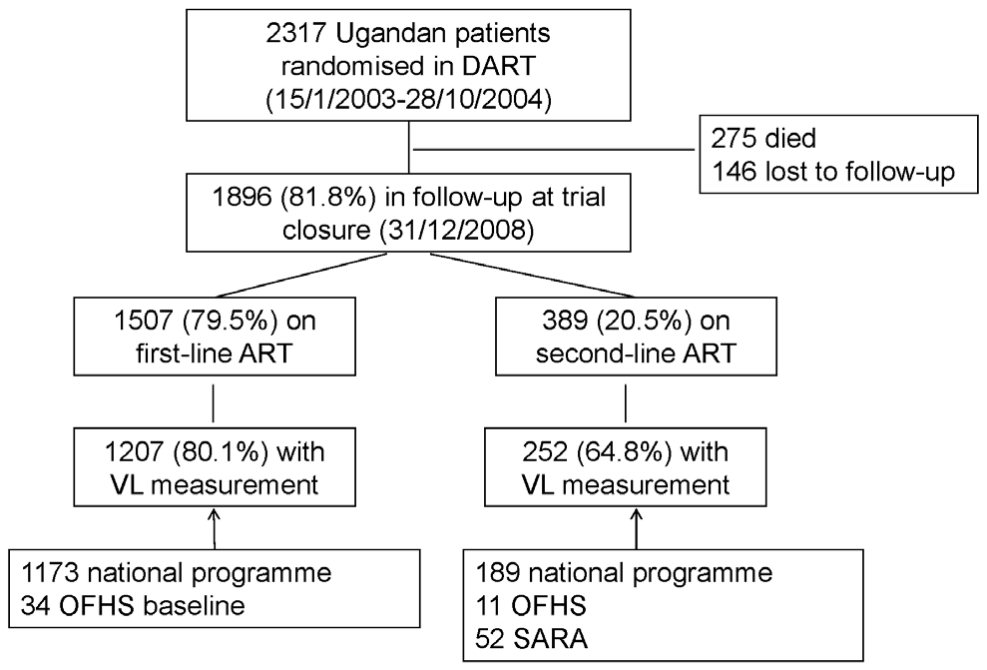
Flow diagram of participants and availability of viral load measurements.

A total of 2317 Ugandan patients were randomised in DART, of whom 275 died (136 within 12 months of trial entry/ART initiation) and 146 were lost to follow-up before the trial closed (Figure 1). The following analyses are based on the remaining 1896 (81.8%) patients, whose characteristics at trial entry are shown in Table 1. Median (IQR, range) follow-up at trial closure was 5.1 (4.7–5.4, 4.2–6.0) years.

**Table 1:** Predictors of switch to second-line ART.

Factor	Number	Switched to second-line ART, n (%)	Unadjusted OR	Adjusted OR (95% CI)^1^
**All patients**	1896	389 (20.5)	-	-
**Monitoring strategy**				P = 0.02
CDM	939	173 (18.4)	1.00	1.00
LCM	957	216 (22.6)	1.29	1.33 (1.04–1.68)
**Age at entry (years)**				P = 0.36
<30	310	76 (24.5)	1.00	1.00
30–34	458	100 (21.8)	0.86	0.86 (0.59–1.23)
35–39	486	84 (17.3)	0.64	0.68 (0.47–0.99)
40–44	340	73 (21.5)	0.84	0.88 (0.59–1.31)
≥45	302	56 (18.5)	0.70	0.79 (0.52–1.21)
**Sex**				P<0.001
Female	1294	229 (17.7)	1.00	1.00
Male	602	160 (26.6)	1.68	1.55 (1.21–1.99)
**Baseline CD4 (cells/mm^3^)**				P<0.001
<50	621	228 (36.7)	7.77	7.38 (4.83–11.28)
50–99	430	82 (19.1)	3.16	2.90 (1.83–4.58)
100–149	442	51 (11.5)	1.75	1.70 (1.04–2.76)
150–199	403	28 (6.9)	1.00	1.00
**Initial ART regimen**				P = 0.013
ZDV/3TC/TDF	1404	315 (22.4)	1.00	1.00
ZDV/3TC/ABC	244	47 (19.3)	0.83	1.29 (0.82–2.03)
ZDV/3TC/NVP	248	27 (10.9)	0.29	0.58 (0.35–0.97)
**Duration of follow-up** 2	-	-	1.03	1.03 (1.00–1.06)

Switches observed over median follow-up of 5.1 years.

1. Multivariate logistic regression analysis, adjusting for all factors listed in Table and study site.

2. Per month.

P-value based on test for heterogeneity or test for trend, as appropriate.

### Switch to second-line ART

The switch rate to second-line ART was low (5.6 per 100 person-years after the first year) and by trial closure only 389 (20.5%, 95% CI 18.7–22.4%) patients had switched to second-line therapy (Figure 1). From multivariate analysis, significant independent baseline predictors of a higher rate of switch to second-line were randomisation to LCM, male sex, lower baseline CD4 count, and a triple NRTI first-line regimen (Table 1). The effect of baseline CD4 count (regardless of monitoring strategy) was particularly strong, showing a clear gradient with the odds of switching to second-line ART over seven-fold higher in the lowest CD4 group (<50 cells/mm^3^) compared with the highest group (150–199 cells/mm^3^).

**Figure 2:**
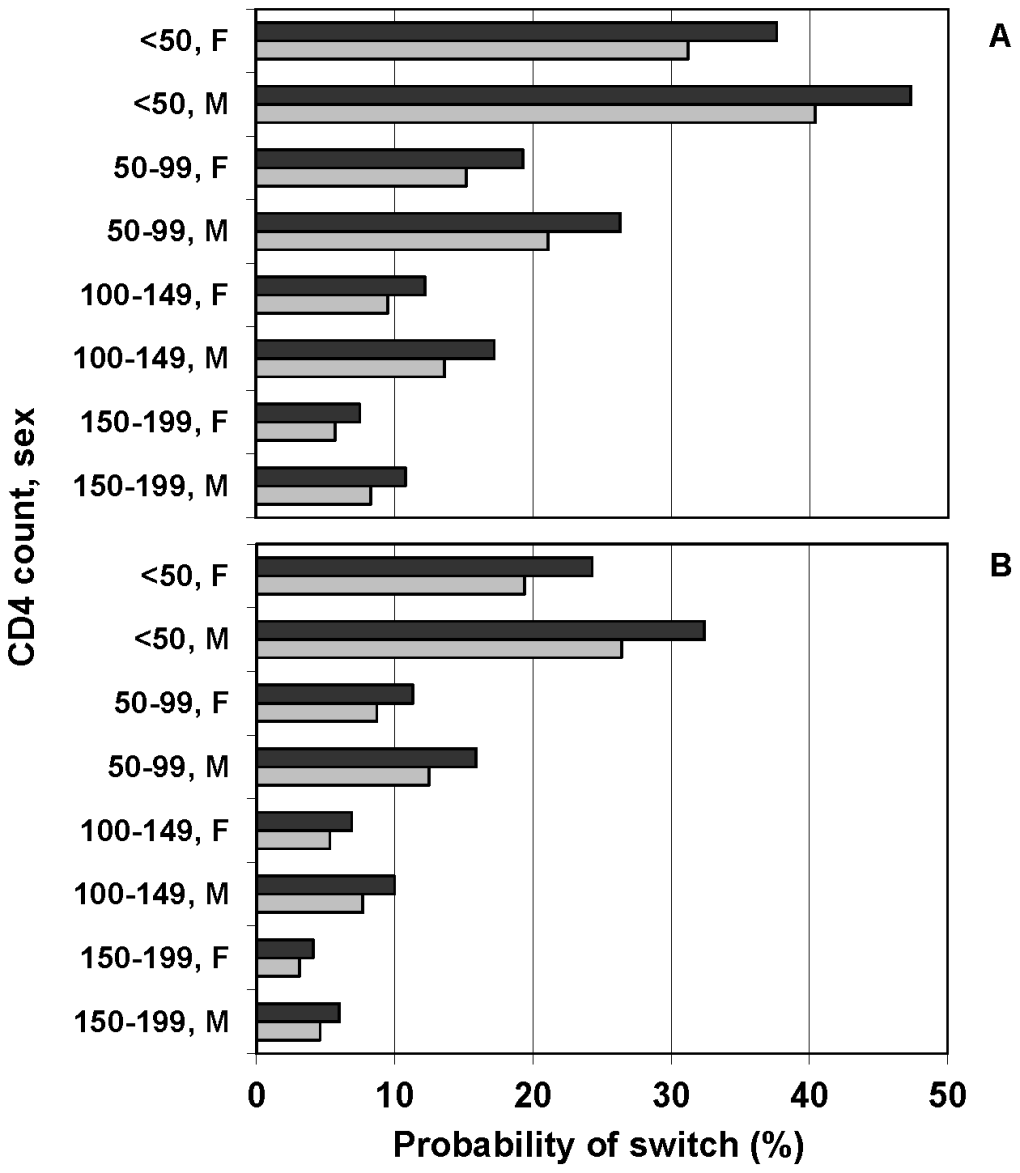
Estimated probability of switching to second-line ART by baseline CD4 count and sex. Legend: Probability of switching by 5 years. A) triple NRTI regimens (B) ZDV/3TC/NVP. Black bars denote LCM group; grey bars denote CDM group.

The predicted probability of switching to second-line ART by five years was calculated for all combinations of significant baseline predictors; estimates are shown separately by initial ART regimen (triple NRTI or NNRTI-based) (Figure 2a and 2b). Apart from patients who initiated ART with <50 CD4 cells/mm^3^, these probabilities were remarkably low, with a minimum value of 3.1% (NNRTI-based ART, CDM, female, 150–199 CD4 cells/mm^3^).

### Viral load at trial closure

The source and number of viral load measurements are shown in Figure 1. A viral load measurement was more frequently available in the Kampala sites (86.3%) than in Entebbe (65.2%) (P<0.001) but was not otherwise associated with any baseline patient characteristic (those listed in Table 1) or with last available CD4 count (result not shown).

### Patients on first-line ART

**Figure 3:**
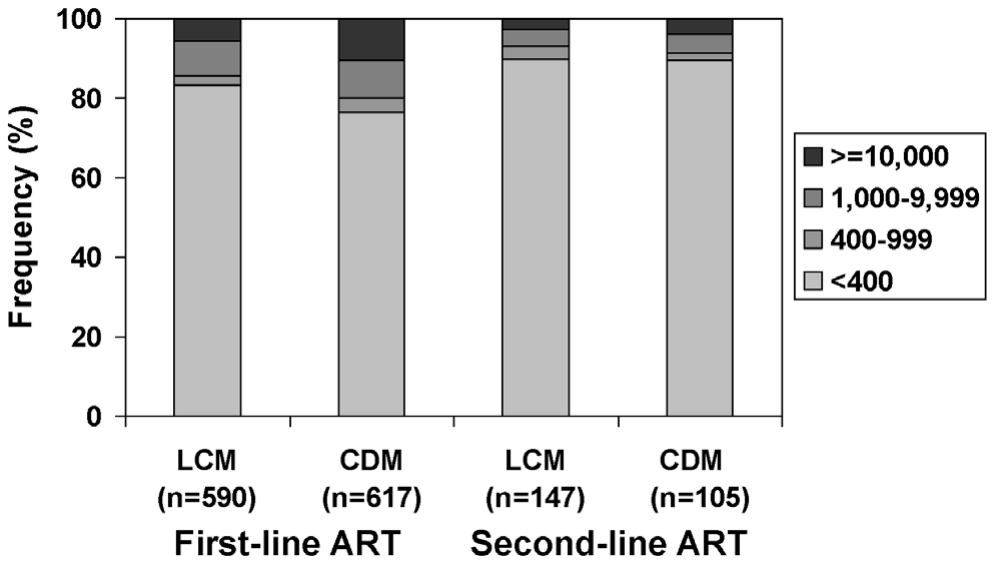
HIV RNA at trial closure by monitoring strategy and line of regimen.

A viral load measurement at trial closure was available on 1207 (80.1%) patients on first-line ART. HIV RNA was <400 copies/ml in 963 (79.8%) patients, 400–999 copies/ml in 37 (3.1%), 1,000–9,999 copies/ml in 110 (9.1%), and ≥10,000 copies/ml in 97 (8.0%). The frequency of viral suppression (HIV RNA<400 copies/ml) was slightly lower in CDM (76.3%) than in LCM (83.4%), a difference of 7.1% (95% CI 2.5–11.5%). This was accompanied by a shift towards a higher proportion of patients with HIV RNA ≥10,000 copies/ml in CDM (10.4% versus 5.6%; difference 4.8%, 95% CI 1.7–7.8%) (Figure 3). Multivariate analysis confirmed the independent effect of monitoring strategy, and better virological outcomes among older patients and among patients who initiated ART with ZDV/3TC/NVP (90.9% suppressed), with little difference between ZDV/3TC/TDF (77.7%) and ZDV/3TC/ABC (78.6%) (Table 2). Borderline significant effects were observed for sex (less suppression among males) and baseline CD4 count (less suppression at lower values). The median HIV RNA level among viraemic (HIV RNA ≥400 copies/ml) patients was 6,310 (IQR 2,040–38,020) copies/ml; the level of viraemia was not associated (P>0.15) with any baseline factor.

**Table 2:** Probability of viral suppression (HIV RNA<400 copies/ml) at trial closure.

	First-line ART		Second-line ART		Both lines of ART
	n/N(%)	Adjusted OR (95% CI)^1^	n/N(%)	Adjusted OR (95% CI)^1^	Probability(%) (95% CI)
**All patients**	963/1207 (79.8)	-	226/252 (89.7)	-	81.8 (79.8–83.7)
**Monitoring strategy**		P = 0.003		P = 0.97	
CDM	471/617 (76.3)	1.00	94/105 (89.5)	1.00	78.8 (75.7–81.6)
LCM	492/590 (83.4)	1.55 (1.16–2.07)	132/147 (89.8)	0.98 (0.40–2.39)	84.8 (82.1–87.2)
**Age at entry (years)**		P<0.001		P = 0.08	
<30	130/182 (71.4)	1.00	39/46 (84.8)	1.00	74.7 (68.7–79.9)
30–34	209/270 (77.4)	1.48 (0.95–2.31)	47/57 (82.5)	0.94 (0.30–2.95	78.5 (73.7–82.6)
35–39	266/326 (81.6)	1.87 (1.20–2.91)	57/60 (95.0)	4.26 (0.95–19.0)	83.9 (80.0–87.2)
40–44	185/225 (82.2)	2.00 (1.23–3.25)	50/53 (94.3)	3.46 (0.72–16.6)	84.8 (80.2–88.5)
≥45	173/204 (84.8)	2.40 (1.43–4.03)	33/36 (91.7)	1.86 (0.41–8.40)	86.1 (81.1–89.9)
**Sex**		P = 0.12		P = 0.29	
Female	688/850 (80.9)	1.00	122/141 (86.5)	1.00	81.9 (79.4–84.2)
Male	275/357 (77.0)	0.78 (0.57–1.06)	104/111 (93.7)	1.71 (0.63–4.59)	81.5 (77.8–84.6)
**Baseline CD4 (cells/mm^3^)**		P = 0.05		P = 0.27	
<50	240/318 (75.5)	0.64 (0.42–0.97)	136/148 (91.9)	1.84 (0.29–11.7)	81.5 (77.8–84.7)
50–99	233/292 (79.8)	0.77 (0.50–1.19)	51/58 (87.9)	0.87 (0.13–5.97)	81.4 (76.9–85.1)
100–149	240/301 (79.7)	0.75 (0.49–1.15)	26/31 (83.9)	1.10 (0.15–7.91)	80.2 (75.6–84.2)
150–199	250/296 (84.4)	1.00	13/15 (86.7)	1.00	84.6 (80.1–88.2)
**Initial ART regimen**		P = 0.001		P = 0.26	
ZDV/3TC/TDF	678/872 (77.7)	1.00	188/207 (90.8)	1.00	80.7 (78.3–82.9)
ZDV/3TC/ABC	125/159 (78.6)	0.90 (0.52–1.53)	21/27 (77.8)	0.40 (0.12–1.32)	78.4 (71.8–83.9)
ZDV/3TC/NVP	160/176 (90.9)	2.78 (1.48–5.20)	17/18 (94.4)	1.70 (0.19–15.1)	91.3 (86.4–94.5)
**Duration of first-line ART** 2	-	0.99 (0.95–1.03)	-	-	-
**Duration second-line ART** 2	-	-	-	1.00 (0.97–1.04)	-

First-line and second-line ART columns show the absolute number (and denominator) of patients with viral suppression (HIV RNA<400 copies/ml). Estimates for both lines of ART are weighted averages accounting for variation in data completeness (see Methods).

Baseline CD4 refers to CD4 at trial entry.

1. Multivariate logistic regression analysis, adjusting for all factors listed in Table and study site.

2. Per month.

P-value based on test for heterogeneity or test for trend, as appropriate.

### Patients on second-line ART

A viral load measurement at trial closure was available on 252 (64.8%) patients on second-line ART, at a median (IQR, range) of 2.3 (1.6–3.0, 0.2–4.8) years after switching. HIV RNA suppression was even higher than among patients on first-line ART: <400 copies/ml in 226 (89.7%) patients, 400–999 copies/ml in seven (2.8%), 1,000–9,999 copies/ml in 11 (4.4%), and ≥10,000 copies/ml in eight (3.2%) (Figure 3). The distribution of HIV RNA was almost identical for the two monitoring strategies. No clear associations with baseline factors were detected, although the power of this analysis is limited by the small number (26) of viraemic patients (Table 2). Specifically, there was no evidence of an effect of duration of second-line ART (P = 0.83).

### Both lines of ART

Combining results on patients on first-line and second-line ART, an estimated 81.8% (95% CI 79.8–83.7%) of patients were virologically suppressed (Table 2). Stratifying by monitoring strategy, the respective values were 78.8% for CDM and 84.8% for LCM. For this and other factors, the rate of viral suppression mainly reflected the patterns observed for patients on first-line ART, the larger of the two groups.

## Discussion

In this cross-sectional analysis of patients who did not receive real-time viral load monitoring, an estimated 82% had HIV RNA<400 copies/ml at an average of five years after ART initiation. An impressive 79% of patients were still on first-line ART at trial closure – thus, the high prevalence of viral suppression was not explained by frequent switching to second-line ART, which included a highly potent boosted PI. These findings are an important contribution to the debate on laboratory monitoring strategies in resource-limited settings.

### Effect of first-line ART regimen and baseline CD4 count

We identified several factors associated with the rate of switching to second-line ART and/or the prevalence of viral suppression among patients who remained on first-line ART. The factors with the strongest effects were monitoring strategy, ART regimen, and baseline CD4 count.

LCM was associated with a significantly higher rate of switch to second-line therapy as a change in regimen could be triggered by a clinical event or a low CD4 count compared with clinical events only in the CDM group. Consequently, among participants on first-line ART, episodes of viraemia are likely to have been more prolonged in CDM, as evidenced by a lower prevalence of viral suppression at trial closure in this group.

A distinctive feature of DART was the use of first-line triple NRTI regimens (received by 87% of patients in the present analysis). The outcomes for the two NRTI regimens, ZDV/3TC/TDF and ZDV/3TC/ABC, were broadly similar. However, this is not a randomised comparison, and the large change in the odds ratio (from 0.83 to 1.29) in the multivariate analysis of switch to second-line ART is suggestive of confounding, underling the need for cautious interpretation. The findings on patients who initiated ART with ZDV/3TC/NVP are likely to be of most interest as this has been one of the most frequently used drug combinations worldwide. Patients on this regimen had a significantly lower switch rate to second-line ART and, consistent with week 48 data, better virological outcomes [23]. Following the use of first-line triple NRTI regimen, the OFHS sub-study data suggest that it may not matter which, if any, NRTIs are included in a second-line regimen comprising a boosted PI and an NNRTI [21]. While numbers were small, excellent viral suppression was also observed in patients who received ZDV/3TC/NVP first-line and thus received only one new class (boosted PI) in second-line.

The effect of baseline CD4 count on the durability of first-line ART was remarkably strong, and up to 97% of patients with a value between 150–199 cells/mm^3^ remained on their first-line regimen at the end of follow-up (Figure 2). The much higher switch rate among participants with a baseline CD4 count less than 50 cells/mm^3^ is a further spur to enter patients early into treatment programmes, before significant immunosuppression has developed. Also, if DART had been conducted in a less clinically advanced population (median CD4 count at ART initiation was 86 cells/mm^3^), it is likely that the overall proportion of patients who switched to second-line ART would have been substantially lower.

### Comparison with other studies

Most information on viral load outcomes in resource-limited settings have been reported from treatment programmes that utilised real-time viral load monitoring. In a large meta-analysis (>25,000 patients), McMahon and colleagues estimated a pooled prevalence of viral suppression of 84% (<300–500 copies/ml, on-treatment analysis) after one year of ART [24]. In a similar analysis of treatment programmes in Africa, Barth and colleagues estimated that 76% and 67% patients on first-line ART were virally suppressed at one and two years respectively, and noted the scarcity of information beyond this time point [26]. The comparatively high prevalence of viral suppression among DART patients on first-line ART (80%) is remarkable for several reasons. First, the estimate pertains to a much later time point (five years); second, the identification of virological failure in real-time in other studies should have prompted more rapid switching to second-line ART; third, the predominant triple NRTI regimens used in DART compare unfavourably with the more common NNRTI-based regimens in terms of virological response.

DART also compares favourably with other published studies that examined response to second-line ART. A recent inclusive meta-analysis of randomised and observational studies in resource-limited settings estimated that the cumulative pooled proportion of patients with virological failure after two years of second-line ART was 27% (based on five studies) and 38% after three years of second-line ART (three studies) [25]. However, there was marked variability between studies, particularly at the three year time point. In contrast, only 10% of DART patients on second-line ART had viraemia at trial closure, an average of 2.3 years after switching. This was despite the fact that 23 (9%) patients were on sub-optimal boosted PI monotherapy at the time of measurement [22].

One plausible explanation for the impressive clinical and virological outcomes in DART is that the clinical care received by DART participants was generally superior to that received in routine treatment programmes, enabling high levels of adherence [26]. That high level care is possible outside the framework of a clinical trial was demonstrated by an independent study of 998 CD4-monitored patients at one of DART study sites (Infectious Diseases Institute), which reported 90% patients with HIV RNA<400 copies/ml after three years of ART, although this site would have gained experience from participation in a trial [27]. However, individual patients can achieve high levels of adherence only if drugs are readily and continuously available (as they were in DART), and there is recognition that drug stock-outs in some resource-limited settings are a key determinant of treatment failure [28], [29].

### Study limitations

Our study has several limitations. First, as discussed above, the high quality clinical care received by DART participants implies that the results may not be widely generalizable [26]. A counter argument is that DART has shown what is achievable, and that widespread viraemia is not an inevitable consequence of not using viral load monitoring, as has been predicted [11], [12]. Second, patients who died or were lost to follow-up before study closure were excluded by definition. However, these represent only 12% and 6% respectively of the patients enrolled, and the high proportion (49%) of deaths which occurred within one year of ART initiation are unlikely to be related to virological failure [30]. Third, while 20% and 35% of patients on first-line and second-line ART respectively lacked a viral load measurement, there was no evidence of a systematic difference between those with and without a measurement, although bias due to the impact of the second-line sub-studies is difficult to exclude. Fourth, as resistance data are not currently available we cannot determine whether virological failure was due to the development of viral resistance or to other factors, such as non-adherence to ART. Finally, our analysis gives a snapshot at a single time-point on average five years after ART initiation. Further testing of DART samples to characterise longitudinal changes in viral load and the evolution of viral resistance are ongoing.

### Clinical and public health implications

In principle, the best evidence on the value of viral load monitoring should come from randomised trials of monitoring strategies that included a viral load monitoring arm (compared with CD4 only or clinical monitoring). Of the three published studies with such a study design to date, none discerned any effect of viral load monitoring on clinical outcomes, but the relatively small study sizes and short follow-up (2–3 years, before most patients experience viral rebound) limits their relevance to the debate on the role of viral load monitoring [31]–[33].

The cost-effectiveness of viral load monitoring has mainly been studied in several computer simulation models [18], [34], [35]. These have produced widely ranging estimates, with determination of cost-effectiveness depending critically on an individual country's willingness-to-pay threshold. A key input parameter in all models is the rate of virological failure; if this is low then a large number of viral load tests need to be performed to identify the few patients in whom a change of ART may be warranted [36], [37]. As discussed above, there is a scarcity of empirical evidence on the rate of virological failure beyond the first two years of first-line ART on which to base this parameter. Our analysis suggests that viral suppression may be more prolonged than has previously been thought.

New WHO guidelines issued in July 2013 include a number of important changes to previous guidelines, including a recommendation that viral load is the preferred monitoring approach to diagnose and confirm ART failure [38]. However, our analysis shows that excellent virological outcomes, as well as immunological and clinical outcomes [20], are possible without routine CD4 and viral load monitoring of patients on ART, provided therapy is delivered in the context of high-quality clinical care. National policymakers need to prioritise between the new WHO recommendations and wider ART coverage, considering available resources and monitoring approaches that can be practicably implemented, to maximise health gains in the population [39].
